# Editorial: Innovations in cataract surgery

**DOI:** 10.3389/fmed.2024.1367577

**Published:** 2024-01-23

**Authors:** Rachid Tahiri, Cati Albou-Ganem, Damien Gatinel, Otman Sandali

**Affiliations:** ^1^Department of Ambulatory Surgery, Avranches Granville Hospital, Granville, France; ^2^Department of Ophthalmology, Vision Clinic of Paris, Paris, France; ^3^Department of Ophthalmology, Rothschild Foundation Hospital, Paris, France; ^4^Department of Ambulatory Surgery, Guillaume de Varye Clinic, Bourges, France

**Keywords:** innovation, cataract, surgery, phacoemulsification, Surgicube^®^, three-dimensional visualization, nanolaser, FLACS

The Research Topic on “*Innovations in Cataract Surgery*” presents the forefront of research and advancements in the field, highlighting remarkable progress in surgical methods, technology, and the enhancement of patient results.

Annually, cataract surgery is performed on 20 million individuals worldwide, marking it as one of the most prevalent surgeries globally (1). The technique of phacoemulsification, introduced by Charles Kelman over five decades ago (2), continues to be the gold standard for cataract surgery (2, 3). This technique has undergone numerous gradual advancements in recent times, leading to surgeries that are less invasive, safer, and facilitate faster visual recovery. These advancements include smaller incision sizes, optimized ultrasound delivery, improved device fluid dynamics, more efficient biometry using Swept source lasers, and integration of augmented reality directly into microscope oculars (4–6).

However, these enhancements appear to be incremental rather than groundbreaking innovations. The introduction of Femtosecond Laser-Assisted Cataract Surgery (FLACS) in the early 2010s initially seemed like a revolutionary leap. Yet, a decade later, it needs to work on becoming the standard due to its higher costs, operational challenges in the operating room, and marginal benefits over traditional phacoemulsification (7). Despite these innovative steps, the question arises whether we have reached a peak in cataract surgery innovation. This Research Topic aims to explore this question, presenting various current and future innovations in cataract surgery.

A comprehensive review of FLACS is presented and offers insights into its technicalities, clinical implications, and cost-effectiveness and underscores its precision and utility in complex cases, though without significantly changing clinical outcomes. Furthermore, a key study in this issue focuses on using 3D visualization in phacoemulsification, demonstrating its role in reducing intraoperative light intensity, decreasing phototoxic damage to the ocular surface, and lessening post-surgery dry eye symptoms. Another interesting research examines the use of the Surgicube, a mobile laminar airflow device, compared to traditional operating rooms. It involved 923 patients, with 448 surgeries in the Surgicube and 475 in standard theaters. Results showed no significant difference in complications, including endophthalmitis, between both groups. The study suggests that using the Surgicube for cataract surgery outside conventional operating rooms is non-inferior to traditional settings, potentially impacting future surgical practices.

This issue also includes comparative studies, such as the one analyzing the differences between ultrasound phacoemulsification and nanosecond laser techniques, with the latter showing better outcomes in reducing endothelial cell loss and corneal damage. Another study compares the traditional ultrasound phacoemulsification and the FemtoMatrix^®^ PhotoEmulsification^®^ technique and highlights FemtoMatrix^®^'s efficiency in lowering effective phaco time and facilitating zero-phaco procedures, particularly in severe cataract cases.

All these studies underscore the ongoing evolution in cataract surgery, pushing the limits of what is currently possible in cataract surgery. This Research Topic displays the latest innovations and encourages further research and development in the sector, aiming to improve patient care and surgical results substantially. We hope you will enjoy reading it.

## Author contributions

RT: Writing – original draft, Writing – review & editing. CA-G: Formal analysis, Methodology, Validation, Writing – review & editing. DG: Formal analysis, Methodology, Validation, Writing – review & editing. OS: Formal analysis, Methodology, Validation, Writing – review & editing.
